# iPSCs: From Bench to Clinical Bed

**DOI:** 10.1155/2016/8367587

**Published:** 2016-09-22

**Authors:** Yujing Li, Changwon Park, Luciano Vellón, Xuekun Li

**Affiliations:** ^1^Department of Human Genetics, Emory University School of Medicine, 615 Michael St, Atlanta, GA 30322, USA; ^2^Department of Pediatrics, Emory University School of Medicine, Atlanta, GA 30322, USA; ^3^Stem Cells Laboratory, Institute of Biology and Experimental Medicine (IBYME-CONICET), Buenos Aires, Argentina; ^4^Institute of Translational Medicine and The Children Hospital, Zhejiang University School of Medicine, Central Building B, 268 Kaixuan Rd, Jianggan District, Hangzhou, Zhejiang 310029, China

Induced pluripotent stem cells (iPSC) have been acknowledged as a milestone in the field of stem cell biology and regenerative medicine, providing an excellent tool to tackle fundamental biological issues regarding reproduction, regeneration, and (de)differentiation at genetic and epigenetic levels, and the valuable cell sources for tissue regeneration, human disease modeling, and drug discovery. Furthermore, iPSC generation with patient-specific somatic cells holds great promise for autologous cell replacement therapy and organ transplantations. Thus, the iPSCs have attracted significant attentions in generation methods, mechanisms of reprogramming, and therapeutical applications. Given a body of achievements in iPSC generation methodology, mechanisms, and clinical application, it is of significance to publish a special issue focused on the topic of iPSCs (from bench to clinical bed). In this special issue, research and review articles are collected, covering the advances in the areas of iPSC generation strategies, molecular mechanisms for reprogramming, iPSC models for human diseases, cell therapy, organ generation, and transplantation.

Starting from the initial discovery of the iPSCs decade ago, significant efforts have been made to develop the protocols with high efficiency and safety to generate clinically relevant cells by employing a variety of (epi)genetic and biochemical approaches. Consequently, some protocols have become practical to significantly enhance the efficiency of iPSCs generation as well as the safety levels of the iPSCs for therapeutic applications. With regard to the progress in the iPSC generation strategy, P. Ji et al. and N. Xie and B. Tang highlight the technical advances in recent years from virus-mediated to virus-free strategies to reactivate the reprogramming factors silenced in the somatic cells and finally give perspective pertaining to the future directions on how to further develop the protocols particularly by high throughput screening to find small molecules and epigenetic modifiers to enhance the iPSC generation efficiency and clinical safety. Although small molecules that have been identified so far have limited effects on enhancement of the somatic reprogramming efficiency, with development of the more efficient screening strategies, it is still expected that appropriate small molecules play important roles in this regard.

Although the human iPSCs (hiPSCs) have been expected to play essential roles in regenerative medicine, the safety of the generated hiPSCs has been a big issue. K.-I. Lee et al. address their own research progress in generation of foot-free and xeno-free iPSCs for clinical therapy purpose by combining the xeno-free/feeder-free culture system and microRNA delivery based mRNA mediated reprogramming. In addition, R. Rungsiwiwut et al. report an important discovery that coculture of hiPSCs combining human foreskin fibroblasts (HFF) with human cord blood-derived serum (hUCS) confers the high pluripotency, differential capacity, and karyotypic stability; even the hiPSCs are cocultured for much extended period, overcoming the instability during the iPSC large scale and long period of culture.

Previously, experiment animals such as mouse and rat serve as the main source for human disease models, while contributing to partial understanding of the pathological mechanisms, but bearing some fatal shortcomings. The discovery of iPSCs opens a new angle for the development of new models to dissect the pathological mechanisms of the human diseases and to discover new strategies for clinical therapy because the iPSCs from patient tissues could be differentiated into the cell types that recapitulate the identical genome of the patient. Although this strategy is still at a very early stage, pilot achievements have been made. In this special issue, N. Xie and B. Tang highlight the recent advances in iPSC models of human diseases with four strategies: (1) directly reprogramming patient somatic cells to iPSCs, (2) generating humanized mouse chimera with iPSCs injection, (3) three-dimensional structured in vitro models, and (4) iPSC-derived minibrains, respectively. In addition, these authors also review the potential challenges encountered in the practice of these strategies. Meanwhile, J. Kang et al. and W. Zhang et al. further review the progress in the iPSC models of human Parkinson's and Alzheimer's diseases in more detailed ways, respectively, and address the challenges and future directions.

With significant improvement on the iPSC generation methods, more and more mysteries behind the molecular mechanisms that regulate the somatic reprogramming at the level of genetics and epigenetics have been uncovered. Several review articles in this special issue highlight the advances in the understanding of the reprogramming mechanisms. At epigenetic levels, S. Hu and G. Shan summarize the global epigenetic remodeling during the somatic reprogramming, particularly the alteration of the long noncoding RNAs (lncRNAs) expression levels. Meanwhile, P. Ji et al. focus on the reprogramming regulation at epigenetic and nonepigenetic levels. Epigenetically, regulations at levels of chromatin, genomic DNA, and histone macroH2A have been proven to play essential roles in the somatic reprogramming regulation. And with discovery of these epigenetic modifiers as well as chromatic remodelers that significantly regulate the somatic reprogramming, some of these regulators have been believed to function as enhancers for the iPSC generation efficiency and potential drugs for therapeutic applications clinically.

Since the iPSCs can be generated from the same patient, recapitulating the whole identical patient genome, the immune rejection encountered in the conventional transplantation can be avoided by applying the iPSCs-based generation of the organs. Thus, the iPSC-based clinical application in regenerative medicine particularly in cell therapy and organ regeneration as well as transplantation has been attracting more and more attentions. In this special issue, A. J. Orqueda et al. highlight the recent breakthrough particularly in the iPSC-derived generation of miniorganoids such as miniature stomach, 3D gut, minilivers, little lungs, building hearts, tiny eyes, and baby brains derived from 3D culturing of the iPSC-based neuroectoderm and these baby brains could lead to further generation of cerebral cortex, ventral telencephalon, choroid plexus, retinal identities, and so forth. These tries have become the pilot in the regenerative medicine and shed light on the in vitro generation of the human organs, opening a new era for the regenerative medicine.

For iPSC-based cell therapy, several review articles in this special issue highlight the recent promising advances. A. J. Orqueda et al. summarize the impact of insulin-producing pancreatic cells, motor neurons, and retinal cells differentiated from iPSCs in either animal models or patients and discuss the application of CRISP/CAS9 technology in target gene mutation corrections for the patient-specific iPSCs. N. Xie and B. Tang summarize the cell replacement strategies and the potential challenges. J. Kang et al. and N. Xie and B. Tang highlight the effects of the iPSCs-derived motor neurons in ischemic stroke rescue in rat PD models.

Since the conventional pharmaceutical drug screening pipeline confers low efficiency, high cost, and extremely low approval rate of the clinically tested drugs, scientists have been trying better alternatives. Because the patient-derived iPSCs could better emulate the real pathological mechanisms, it is logical to speculate that the patient-derived iPSCs-based drug screening could provide a better platform efficiently and identify the candidates from the large number of chemical compounds. In addition, the iPSCs-based platforms could more efficiently assess cell/tissue specific off-target effects and toxicities. In this special issue, N. Xie and B. Tang summarize the progress made in the iPSCs-mediated drug screens and address the challenges.

In summary, by highlighting the recent advances in iPSC research from methodology to the mechanisms and from bench experiments to the clinical applications, the authors of the research and review articles in this special issue hope that they contribute a shortcut for the readers to get the related information easily, who are dedicated in the research field of iPSC and regenerative medicine.

*Yujing Li* *Yujing Li* *Changwon Park* *Changwon Park* *Luciano Vellón* *Luciano Vellón* *Xuekun Li* *Xuekun Li*

